# Digital gamification and serious games for preventing e-cigarette use in adolescents: A systematic review without meta-analysis (SWiM)

**DOI:** 10.18332/tid/214070

**Published:** 2026-07-16

**Authors:** Siti Idayu Hasan, Ruthashini R Selvasingam, Farizah Mohd Hairi

**Affiliations:** 1 Department of Social and Preventive Medicine, Faculty of Medicine, University of Malaya, Kuala Lumpur, Malaysia; 2 Centre for Population Health, Department of Social and Preventive Medicine, Faculty of Medicine University of Malaya, Kuala Lumpur, Malaysia; 3 Nicotine Addiction Research and Collaboration Group, University of Malaya, Centre for Addiction Science Studies, Kuala Lumpur, Malaysia

**Keywords:** digital gamification, e-cigarette, vape, adolescents, serious game

## Abstract

**INTRODUCTION:**

Adolescent e-cigarette use has reached alarming levels worldwide, creating a major public health concern. Digital gamification and serious games have emerged as an innovative approach to engage youth in e-cigarette prevention through interactive and immersive technologies. However, existing reviews on gamification and serious games for health have not specifically examined theoretical frameworks or behavioral outcomes in digital gamification for e-cigarette prevention among adolescents. This systematic review aims to assess the effectiveness of digital gamification and serious game interventions for e-cigarette prevention in adolescents and to identify the theoretical foundations underlying these interventions.

**METHODS:**

Eight electronic databases were searched (MEDLINE, CINAHL, Psychology and Behavioral Sciences Collection, Education Research Complete, Scopus, PubMed, Web of Science, and Google Scholar) up to 25 October 2025. Inclusion criteria covered randomized and nonrandomized studies involving adolescents (aged 10–19 years) using digital gamification and serious games for vape prevention or cessation. Study quality was assessed and a narrative synthesis was conducted.

**RESULTS:**

Twelve studies met the inclusion criteria. Most reported improvements in knowledge, risk perception, and self-efficacy among adolescents. Theoretical frameworks included the Theory of Planned Behavior, Social Cognitive Theory, and the Attention, Relevance, Confidence, and Satisfaction motivation model. Study quality ranged from weak to strong.

**CONCLUSIONS:**

Digital gamification and serious games show promise in supporting e-cigarette prevention among youth. Interventions grounded in behavioral theory and incorporating social and motivational elements are most effective.

## INTRODUCTION

The rise in e-cigarette use among adolescents has become a public health concern. With e-cigarettes marketed as safer alternatives to traditional tobacco products, the growing popularity among youth has led to alarming usage rates, which raise questions about their long-term health implications^1^. Approximately 20% of high school students reported using e-cigarettes in the past month, highlighting the urgent need for effective prevention and cessation strategies. A significant rise in e-cigarette use among Malaysian adolescents is seen in the National Health and Morbidity Survey (NHMS) in 2022^2^, with 14.9% of teens aged 13–17 years using e-cigarettes or vape products, an increase from 9.8% in 2017^3^. This increase is particularly glaring among males, with 23.3% using e-cigarettes in 2022 compared to 6.2% in females. In addition, the Global Adult Tobacco Survey in 2023 found that e-cigarette use among Malaysians aged 15–24 years rose from 1.1% in 2011 to 8.6% in 2022^4^.

E-cigarettes include a diverse group of devices that allow users to inhale an aerosol, which typically contains nicotine, flavorings, and other additives. These devices are referred to by a variety of names, including ‘e-cigs’, ‘e-hookahs’, ‘mods’, ‘vape pens’, ‘vapes’ and ‘tank systems’^5^. Nicotine intake harms the developing brain (i.e. negative impact on attention and cognition) and renders youth vulnerable to other substance use. The use of e-cigarettes has also been associated with worsened lung health and mental well-being^6-9^. A recent study found that dual users were eight times more common in the cases with lung cancer than the control subjects, and the risk of developing lung cancer was four times higher among those dual users than those who only smoked^10^. Nevertheless, the potential development of nicotine addiction and potential health risks is particularly concerning for non-smokers choosing to use e-cigarettes and expose themselves to risk unnecessarily.

Despite the short- and long-term impact, the rise in vaping seen throughout the world, including New Zealand, Australia, the United Kingdom and the United States, driven by several factors. Individual risk factors include use by peers, curiosity desire to experiment, a perceived lack of harmful effects compared to traditional cigarettes^11^ and a history of tobacco product use. Leading environmental risk includes exposure to vaping-related marketing campaigns and easy access to vaping products at low cost. Moreover, vape has been marketed as an effective smoking cessation aid, appealing flavors (‘Fruit Medley’ and ‘Creme Brule’)^12,13^ and attractive marketing of a product with a sleek, trendy design, particularly to youth^14^. Lack of effective regulatory measures, such as restricting advertising, banning flavored products (except tobacco), and limiting nicotine concentrations, can significantly influence youth vaping behavior. These misconceptions and misinformation further exacerbate this behavior.

In this context, serious games, digital gamification, and game-based learning (GBL) have emerged as promising tools to improve learning, motivation, and engagement in health promotion. Gamification is defined as ‘the use of game elements in non-game contexts’^15^ including points, badges, leaderboards, and challenges, to stimulate gameful experiences and reinforce desired behaviors. Serious games are fully developed digital games designed for purposes beyond entertainment such as education, health promotion, or training where gameplay itself constitutes the learning experience. The goal of game-based learning (GBL) is to accomplish predetermined learning objectives by means of interactive problem-solving, feedback, and reflection in game settings. Despite their conceptual differences, the main objective of these strategies is the same: to use play-based motivation to accomplish significant learning and behavior change. Because of these concepts, gamification is particularly appropriate for adolescents who are used to online social comparison dynamics and digital interaction.

An increasing body of evidence supports the effectiveness of gamification and serious games in changing health behaviors. A meta-analysis of 77 studies by Sailer and Homner^16^ found significant positive effects of gamification on behavioral outcomes such as engagement, adherence, and performance, especially when interventions incorporated meaningful feedback and social elements. Similarly, systematic reviews have shown that serious games and GBL interventions improve health-related behaviors, including physical activity, dietary habits, and substance use prevention^17,18^. Serious digital games have successfully reduced smoking initiation, boosted refusal self-efficacy, and increased risk perception in youth^19^. Gamified cognitive-behavioral interventions for mental health have been proven to enhance coping skills, emotional regulation, and treatment adherence^20^. Overall, these findings affirm that game-informed digital interventions are not merely entertaining but can generate measurable behavioral change through immersive, experiential learning mechanisms. Alongside being enjoyable. However, evidence directly targeting e-cigarette prevention among adolescents remains sparse and fragmented. Existing studies vary widely in design quality, theoretical foundation, and outcome measures, limiting generalizability. Moreover, few interventions have been developed or validated within low- and middle-income country contexts such as Malaysia, where cultural and regulatory factors influence youth engagement with vaping content.

Given these gaps, a systematic synthesis of digital gamification and serious-game interventions targeting e-cigarette prevention is both timely and critical. Such evidence would align with the World Health Organization’s (WHO) Tobacco-Free Initiative and the global call for innovative, technology-driven strategies to curb nicotine use among youth^21^. Understanding how game mechanics, behavioral theories, and digital learning frameworks intersect to influence adolescents’ knowledge, attitudes, and behavioral intentions is essential for developing scalable interventions that resonate with digital-native populations. By bridging insights from behavioral science, educational technology, and tobacco control, this review provides a comprehensive overview of how gamified and serious-game approaches can serve as catalysts for sustainable health behavior change. Therefore, this study aimed to systematically review and critically appraise existing evidence on digital gamification, serious games, and game-based learning interventions designed to prevent e-cigarette use among adolescents, with a focus on their theoretical foundations, intervention characteristics, and effectiveness in influencing vaping-related behaviors.

## METHODS

This systematic review adhered to the 2020 Preferred Reporting Item for Systematic Review and Meta-analysis (PRISMA) guidelines^22^ and its extension Synthesis Without Meta-analysis (SWiM)^23^. The review protocol was registered in the International Prospective Register of Systematic Reviews (PROSPERO) (Registration number: CRD42024582882) before the study convened.

### Eligibility criteria

The study’s selection criteria were structured using the Population, Intervention, Comparison, Outcome, and Study design (PICOS) framework. This review focuses on adolescents aged 10–19 years as the target population. The interventions of interest include digital gamification and serious games designed to prevent or cease e-cigarette use. Studies with or without a comparison group are included. The primary outcomes assessed are vaping prevention and cessation among adolescents. Randomized Studies of Interventions (RSIs) (RCTs, Cluster RCTs) and Nonrandomized Studies of Interventions (NRIs) were included. Studies that were not published in the English language, editorials, reviews, conference proceedings, those unrelated to the population of interest, and those lacking digital gamification and serious game interventions were excluded.

### Database and search strategies

The articles were retrieved from electronic databases MEDLINE, CINAHL, Psychology and Behavioral Sciences Collection, Education Research Complete, Scopus, PubMed, Web of Science and Google Scholar. Additionally, grey literature and reference lists of included studies were utilized to identify further relevant articles. A comprehensive search was performed in the databases using the identified keywords and MeSH terms employing AND and OR to combine the search terms as follows: [(electronic nicotine delivery* OR vape* OR e-cig*) AND (child* OR Adolescence* OR teen* OR youth*) AND (prevent* OR cease*) AND (gamification* OR game elements* OR serious games* OR video games* OR digital games* OR gamification in education* OR gamified learning* OR game-based learning*)]. See the Supplementary file for the full search strategy which was applied systematically to each database from the date of database inception to October 2025. All retrieved records were exported to EndNote.

### Study screening and selection

**Figure 1:**
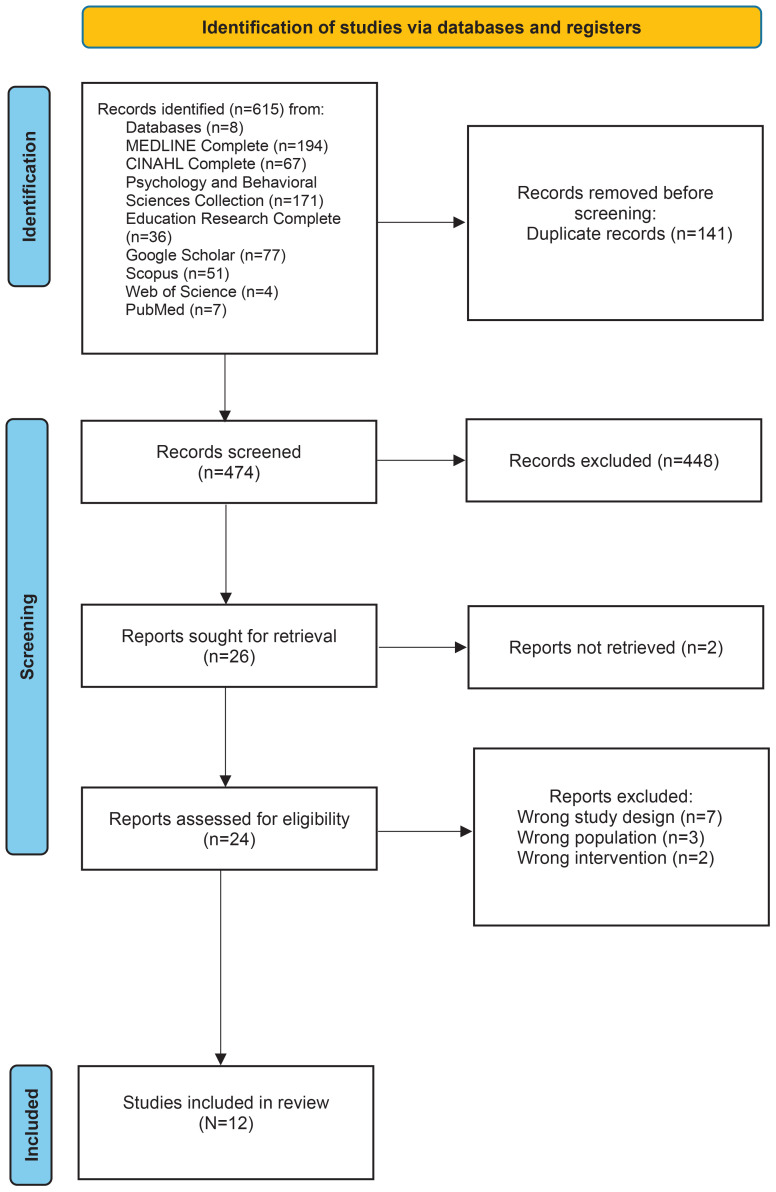
PRISMA flow diagram of selection of studies

Studies retrieved were imported to Rayyan software^24^ where duplicate articles were removed following the deduplication process. Titles and abstracts were screened, with two independent reviewers (SIH and RR) assessing studies based on predetermined eligibility criteria. A full-text review was conducted by two independent reviewers (SIH and RR) for all included studies. Forward and backward searching of these selected studies was then performed on the Web of Science to further supplement the overall search. The study selection process is documented in the PRISMA flow diagram (Figure 1).

### Data extraction

Two reviewers (SIH and RR) independently extracted data using a structured data extraction form that included author, publication year, study design, and population demographics. Relevant theories, characteristics of gamification and serious game interventions, measures of vaping initiation and cessation, and other outcomes such as knowledge, attitude, perception, and self-efficacy regarding vaping were extracted. All relevant outcomes aligned with the defined outcome domains were systematically identified and documented from each included study. Any discrepancies were resolved through discussion.

### Assessment of study quality

Study quality was independently evaluated by two reviewers using the Effective Public Health Practice Project (EPHPP)^25^, a validated tool recommended for assessing the quality of different study designs. It consists of six criteria: selection bias, allocation bias, control of confounders, blinding of outcome assessors, data collection methods, and withdrawals, and dropouts. Reviewers were asked to rate each criterion as ‘weak’, ‘moderate’, or ‘strong’. Any discrepancies were resolved through discussion.

### Strategy for data synthesis and synthesis without meta-analysis

Data was extracted from individual studies to describe key findings. The SWiM method was undertaken as a meta-analysis was not possible due to outcome and methodological heterogeneity across the included studies. The study populations varied considerably with respect to demographic characteristics and inclusion criteria, limiting the comparability of findings. Furthermore, there was marked inconsistency in the way outcomes were defined and reported. Studies employed a wide range of statistical approaches, including mean differences and odd ratios. Additionally, some studies reported means with associated p-values, while others provided descriptive analyses. The variability in outcome metrics, statistical frameworks, and population characteristics precluded the generation of a meaningful pooled estimate of effect size and variance. As such, vote counting for direction of effect was undertaken to describe key findings, keeping in mind contextual differences across studies. The synthesis involved nine key steps: 1) Clear justification for grouping of studies based on relevant characteristics; 2) Standardization of metrics for each outcome; 3) Details of alternative synthesis method employed; 4) Study prioritization criteria; 5) Heterogeneity investigation; 6) Certainty of findings assessment; 7) Visualization of data which clearly link studies to synthesis findings; 8) Synthesis of findings reported in relation to the review question, including contributing studies and certainty assessments; and 9) Limitations of the synthesis method and critically evaluated data grouping. A formal GRADE assessment was not undertaken due to the methodological limitations inherent to the synthesis approach used. This review employed SWiM framework, with findings presented according to the direction of effect rather than pooled effect estimates. In the absence of quantitative summary measures (e.g. risk ratios, mean differences, or confidence intervals), key domains required for GRADE, such as imprecision, consistency, and magnitude of effect could not be adequately assessed. Moreover, substantial heterogeneity in study populations, outcome definitions, and statistical reporting precluded meaningful evaluation of the overall certainty of evidence. Given that GRADE assessments rely on a transparent appraisal of both statistical precision and effect size, the nature of the available data limited the feasibility and validity of such an appraisal. As such, a formal GRADE rating was not applied.

## RESULTS

### Study selection

Database search yielded 615 records (Figure 1). Following removal of 141 duplicates, 474 publications were eligible for abstract and title screening. This resulted in 26 studies being sought for retrieval and 24 were eligible and screened by their full text. The main reasons for excluding studies were wrong study design, intervention and population. Out of these, 12 studies were included in the review.

### Study characteristics

Twelve studies included four Randomized Studies of Interventions (RSIs)^26-29^ and eight Non-Randomized Studies of Interventions (NRSIs) (Table 1). The RSIs consisted of two three-armed cluster RCTs^26,27^ and two two-armed cluster RCTs^29,30^. The NRSIs included one controlled clinical trial^31^ and seven one-group quasi-experimental studies^32-38^. Studies were conducted in the USA^28,30,33-35,37^, Finland^27,29^ and Taiwan^31^, with participants aged 10–18 years. Interventions included digital health games, VR-based, and web-based e-cigarette prevention programs. Sample sizes ranged from 16 to 560 adolescents.

**Table 1:** Study characteristics of gamified and serious game interventions for youth e-cigarette prevention

*Study*	*Country*	*Study design*	*Setting and population*	*n*	*Intervention type*	*Gamification /* *serious game*	*Measurement points*
Khalil et al.^26^ 2024	USA	Cluster-randomized comparative trial	4 After-school sites Youth (11–18 years)	45 (ASPIRE 16; Storm-Heroes 29)	ASPIRE (web) vs Storm-Heroes (board game)	Gamification	Baseline, immediate post, about 1.5-month follow-up
Nyman et al.^27^ 2024	Finland	Cluster RCT (school-level randomization)	Primary schools (grades 4–6)	292 (Fume + Debrief) vs 293 (Control)	Fume + debrief vs Control	Serious game	Baseline, 2 weeks, 3 months
Miller et al.^37^ 2023	USA	Quasi experimental	Appalachian Career Training in Oncology (ACTION) Program (15–16 years)	16	iCANendthetrend Program	Gamification	Baseline, immediate, 4 weeks follow-up
Miley et al.^36^ 2022	USA	Quasi experimental	33 Individual groups (one- or three-lesson delivery) (4th-12th grade)	383	#iCANendthetrend	Gamification	Baseline, immediate, 4 weeks follow-up
Greene et al.^35^ 2021	USA	Randomized controlled trial	4-H youth club members (12–17 years)	310	REAL Media	Gamification	Baseline, immediate, 3-and 9-month follow-up
Guo et al.^31^ 2021	Taiwan	Prospective observational (no control)	High school Taipei City	130	Educational VR	Gamification	Pretest, gameplay session (about 35–45 min), posttest (same visit)
Weser et al.^30^ 2021	USA	Non-equivalent control-group design	3 Middle schools in a Connecticut district Adolescents (11–14 years)	287 (Baseline intervention 150, control 129; post 1 week 142 vs 126; 3-month 142 vs 123; 6-month 110 vs 91)	Invite Only VR vs Control	Serious game	Baseline, post 1 week, 3-month, 6-month
Weser et al.^34^ 2021	USA	Single-group quasi experimental	Adolescents from a local high-school afterschool sports program (13-16 years)	38	Invite Only VR	Serious game	Pre-game, post-game over 2 sessions (1–5 days apart)
Bteddini et al.^28^ 2023	USA	Randomized, 3-arm pilot trial	Rural Florida 4-H youth (11–17 years)	Baseline 76 (CATCH My Breath 28; smokeSCREEN 21; Control 27) Post 60 (CATCH 24; smokeSCREEN 13; Control 23)	CATCH My Breath vs smokeSCREEN vs Control	Gamification	Pre and immediate post-intervention survey
Hieftje et al.^32^ 2019	USA	Real-world quasi-experimental single-group pre–post	Schools and afterschool programs in Rhode Island, Massachusetts, California, and Arizona (10–16 years)	560	smokeSCREEN	Serious game	Pre, post (same program window; gameplay about 1–2 h)
Pentz et al.^33^ 2019	USA	Single-group quasi experimental	Adolescents from community afterschool programs (11–14 years)	80	PlayForward: smokeSCREEN	Serious game	Pre, post program (about 4 weeks)
Parisod et al.^29^ 2018	Finland	Single-blind three-arm cluster RCT	Municipal schools (10–13 years)	151 (Fume 61; Website 47; Control 43)	Fume (game) vs Website (non-gamified content) vs Control	Serious game	Baseline, 2-week follow-up

### Theories

This review identified diverse theoretical frameworks applied in recent studies, including both single and multi-theory approaches (Table 2). Nyman et al.^27^ and Parisod et al.^29^ applied Bandura’s Self-Efficacy Theory to examine self-efficacy in behavior. Bteddini et al.^28^, Weser et al.^30,34^, Hieftje et al.^32^ and Pentz et al.^33^ utilized the Theory of Planned Behaviour^38^ and Social Cognitive Theory^39^ to explore behavioral intentions and cognitive processes. Guo et al.^31^ applied the ARCS model of motivation, focusing on attention, relevance, confidence, and satisfaction. Khalil et al.^26^ integrated Social Learning Theory, the Health Belief Model, the Transtheoretical Model, and Empowerment Theory to investigate health behaviors.

**Table 2:** Intervention characteristics and game design features of included studies

*Study*	*Theory/framework*	*Intervention*	*Format/ platform*	*Key game design*
Khalil et al.^26^ 2024	Social cognitive theory and gamification design principles	Storm-Heroes (+ ASPIRE integration)	Hybrid tabletop + web-based modules	Cooperative gameplay with acting, drawing, and trivia challenges; team missions with points and rewards; integration of ASPIRE digital content; group role-play and reflection activities.
Nyman et al.^27^ 2024	Social cognitive theory and self-efficacy model	Fume	Tablet-based digital game	Interactive peer-based challenges; scenario-based choices; animated feedback; progress indicators; replayable levels reinforcing refusal skills.
Miller et al.^37^ 2023	Health communication and peer-to-peer advocacy model	#iCANendthetrend (ACTION Program)	Online Zoom-based training with interactive tools	Near-peer-led sessions incorporating Kahoot challenges and polls; virtual discussions; reflective activities; interactive mentorship format.
Miley et al.^36^ 2022	Diffusion of innovations and social influence theory	#iCANendthetrend	Virtual classroom/online delivery	Peer-led virtual sessions using Kahoot quizzes, live polls, and interactive chat; gamified reinforcement of e-cigarette prevention content.
Greene et al.^35^ 2021	Message design logic and media literacy theory	REAL Media	Web-based interactive e-learning	Media-literacy training through drag-and-drop activities, mini-quizzes, and ‘create-your-own-message’ challenges; progress feedback after each level.
Guo et al.^31^ 2021	Social influence theory	Educational VR	VR-based educational simulation	3-D interactive educational experience; gamified peer-learning tasks; animated scenarios; immediate feedback and progress tracking.
Weser et al.^30^ 2021	Theory of planned behavior and social cognitive theory	Invite Only VR	Virtual reality (Oculus Go)	Immersive 3-D narrative; branching conversations with avatars; voice and gesture interaction; progress feedback; peer-pressure simulation scenarios.
Weser et al.^34^ 2021	Extended Parallel Process Model (EPPM)	CATCH My Breath vs smokeSCREEN vs Control	Mobile/tablet	Scenario-driven missions; e-cigarette risk simulations; educational minigames; feedback messages; badge rewards; interactive quizzes.
Bteddini et al.^28^ 2023	Theory of planned behavior	Invite Only VR (Prototype Evaluation)	Virtual reality (Oculus Go)	Same core design as above; refined storyline and interface; adaptive feedback; usability and engagement evaluation.
Hieftje et al.^32^ 2019	Theory of planned behavior and social cognitive theory	smokeSCREEN	Web-based serious game	Interactive decision-making scenarios; dialogue-driven storytelling; mini skill-based games; feedback and reflection; personalized progress dashboard.
Pentz et al.^33^ 2019	Social cognitive theory and health behavior model	PlayForward: smokeSCREEN	Tablet-based game	Story-based missions; avatar customization; social scenarios; reward and point system; virtual feedback reinforcing healthy choices.
Parisod et al.^29^ 2018	Health literacy framework and cognitive theory of multimedia learning	Fume (Prototype)	Mobile health game	Storyline-based progression; visual storytelling; quizzes and missions; progress tracking; multimedia feedback; points and badges.

### Intervention

Intervention implementation varied by setting, duration, frequency, delivery mode, and follow-up. Interventions were delivered to individual, school, afterschool settings^26,33^ or combinations thereof (Table 2)^32^. Delivery modes included in-person (others) and online/remotely^28,32^. Session lengths ranged from 15 minutes to one hour, with one to five sessions over up to five weeks. Follow-up assessments were conducted up to nine months of post-intervention^30^. Delivery methods include web-based video games^28,29,32,33^, VR headsets^30,31,34^, head-mounted displays with joysticks^31^, or tablet/smartphones. Curriculum content varied by module and intervention type.

### Effectiveness of gamification approach in e-cigarette prevention – SwiM synthesis

Effect sizes were synthesized across six outcome domains derived from 12 included studies involving gamified and serious game interventions. Reported effects were expressed using standardized metrics, including standardized mean differences (d), standardized beta coefficients (β), and mean change scores (ΔMean). The overall direction of effect was coded as positive (↑) or neutral, consistent with the SWiM framework (Table 3).

**Table 3:** Effect size summary by outcome domain

*Outcome domain*	*Studies contributing*	*Reported effect*	*Effect* *direction*	*SWiM narrative summary*
**Knowledge**	Khalil et al.^26^ (Gamification) Miley et al.^36^ (Gamification) Guo et al.^31^ (Gamification) Weser et al.^30^ (Serious Game) Weser et al.^34^ (Serious Game) Bteddini et al.^28^ (Gamification) Hieftje et al.^32^ (Serious Game) Pentz et al.^33^ (Serious Game) Parisod et al.^29^ (Serious Game)	Khalil β=0.53 (p=0.01); Weser^34^ d=0.95; Miley OR =5.64 (p<0.001)	↑ (10 of 12)	Knowledge improved across nearly all studies; effects moderate–large (d about 0.5–0.9); both gamified and serious games effective for tobacco education.
**Perceived harm/risk**	Khalil et al.^26^ (Gamification) Pentz et al.^33^ Weser et al.^30^ (Serious Game) Weser et al.^34^ (Serious Game) Bteddini et al.^28^ (Gamification) Hieftje et al.^32^ (Serious Game)	Khalil β=0.40 (p=0.02); Weser^34^ d=0.53 (p=0.002); Miley perceived danger ↑ (p<0.001)	↑ (6 of 8)	Most interventions increased perceived risk or harm of vaping; effect sizes small–moderate but consistent across formats.
**Refusal/self-efficacy**	Nyman et al.^27^ (Serious Game) Green et al.^35^ (Gamification) Miller et al.^37^ (Gamification) Wesser et al.^34^ (Serious Game) Weser et al.^30^ (Serious Game) Parisod et al.^29^ (Serious Game)	Nyman β=0.40 (95 % CI: 0.03–0.77, p=0.03); Miley ΔMean ↑ (p<0.01) Greene β=0.13 (p<0.01); Miller t=2.02 (p=0.062)	↑ (4 of 5)	Self-efficacy to refuse vaping/smoking showed small–moderate improvement in both gamified and serious game formats.
**Behavioral intentions/susceptibility**	Bteddini et al.^28^ (Gamification) Guo et al.^31^ (Gamification) Weser et al.^30^ (Serious Game) Weser et al.^34^ (Serious Game) Hieftje et al.^32^ (Serious Game) Pentz et al.^33^ (Serious Game)	Weser^34^ t(35)=2.14, p=0.039 (↓ likelihood to use, d=1.27); others not significant	↑ (3) – (2)	Intentions to use e-cigarettes generally declined; VR and peer-led programs showed protective trends, though effects vary by follow-up.
**Social norms/beliefs**	Bteddini et al.^28^ (Gamification) Hieftje et al.^32^ (Serious Game) Greene et al.^35^ (Gamification) Weser et al.^30^ (Serious Game) Pentz et al.^33^ (Serious Game)	Greene peer disapproval ↑ β=0.12 (p<0.01); others not significant	↑ (2 of 3)	Media-literacy and interactive programs strengthened anti-vaping norms; VR serious games showed neutral effects.
**Acceptability/engagement**	Hieftje et al.^32^ (Serious Game) Green et al.^35^ (Gamification) Weser et al.^34^ (Serious Game) Miley et al.^36^ (Gamification) Guo et al.^31^ (Gamification) Weser et al.^30^ (Serious Game)	Miley >90 % positive feedback, OR =6.56 (p<0.001)	↑ (5 of 5)	Youth rated both VR and gamified programs as highly engaging and enjoyable; strong support for feasibility and acceptability in school settings.

↑ Improvement or favorable change. ↓ Unfavorable change. – No change. p: probability value. d: Cohen’s d (standardized effect size); β: standardized regression coefficient. t: t-statistic (test of mean difference). ΔMean: mean change scores

### Knowledge outcomes

Seven out of ten studies reported significant improvements in knowledge following exposure to gamified or serious game interventions. Reported effect sizes ranged from small to large (d about 0.5–0.9), with metrics such as β=0.41 (p<0.05) and d=0.95.

### Perceived harm and risk

Five out of six studies observed increases in perceived risk or danger associated with vaping or tobacco use. Reported standardized coefficients ranged from β=0.42 to d=0.53, with statistically significant gains in risk perception (p<0.001). These effects were consistent across both gamification and serious game modalities.

### Refusal/self-efficacy

Three out of six studies demonstrated improvements in self-efficacy. Reported values included β=0.40 (95% CI: 0.03–0.77, p<0.05) and significant mean increases (ΔMean ↑, p<0.01). These findings were consistent across studies employing serious games and gamified applications.

### Behavioral intentions and susceptibility

One study reported significant decreases in behavioral intentions to use e-cigarettes (β= -0.25, p<0.01; intent ↓, p<0.05), while two studies showed non-significant results. Overall, the trend indicated a reduction in susceptibility toward e-cigarette initiation.

### Social norms and beliefs

One out of five studies assessing social and normative beliefs reported small yet statistically significant effects (e.g. peer disapproval β ↑, p<0.01). One study found no significant differences between groups.

### Acceptability and engagement

One out of seven studies evaluating acceptability and engagement reported high satisfaction levels, with ≥80% of participants rating the programs as enjoyable or engaging. Reported engagement rates exceeded 90% in gamified formats, with session ratings indicating strong user acceptance and usability across both virtual and hybrid delivery modes.

### Vote count

A total of twelve studies were included in the vote count synthesis, examining outcomes related to knowledge, perceived risk, refusal self-efficacy, intentions, susceptibility, social norms and beliefs, and acceptability or engagement. Across studies, most interventions demonstrated favorable effects in multiple psychosocial domains (Table 4).

**Table 4:** Vote count summary by study

*Study*	*Knowledge*	*Perceived risk*	*Refusal/* *self-efficacy*	*Intentions*	*Susceptibility*	*Social norms/* *beliefs*	*Acceptability/* *engagement*
Khalil et al.^26^ 2024	↑*	↑*					
Nyman et al.^27^ 2024			↑*				
Miller et al.^37^ 2023			↑				↑
Miley et al.^36^ 2022	↑**						↑*
Greene et al.^35^ 2021			↑*			↑*	↑
Guo et al.^31^ 2021	↑			↑			↑
Weser et al.^30^ 2021	↑*	↑*	–	↑		–	↑
Weser et al.^34^ 2021	↑**	↑**	–	↑*			↑
Bteddini et al.^28^ 2023	↑*	↑*		–	–	–	
Hieftje et al.^32^ 2019	↑*	↑*		–		↑	↑
Pentz et al.^33^ 2019	↑	↑		–		–	
Parisod et al.^29^ 2018	↑		–				

↑ Favorable effect.

– No change.

↓ Unfavorable effect.

* p<0.05.

** p<0.001.

### Knowledge and perceived risk

Ten studies evaluated knowledge outcomes, and every study (100%) reported a favorable increase in post-intervention. Similarly, perceived risk was examined in six studies with all showing facvourable effects and one (10%) reporting no change. These outcomes were measured using validated self-report instruments to assess understanding of e-cigarette harms and perceived susceptibility to nicotine dependence. Collectively, these results demonstrate a consistent pattern of cognitive enhancement across interventions that incorporate educational or game-based content.

### Refusal self-efficacy and behavioral intentions

Refusal self-efficacy was measured in six studies, three (50%) of which demonstrated favourable improvement. Three studies (50%) reported neutral outcomes. Behavioral intention outcomes (n=6) showed greater variability: three studies (50%) demonstrated reduction in vaping, three (50%) indicated no favourable change. These mixed patterns reflected differences in intervention duration, exposure frequency, and measurement scales across trials.

### Susceptibility and social norms

Six studies reported on susceptibility outcomes, of which four (67%) showed no change and two (33%) showed unfavorable results. In contrast, social norms and belief-related constructs were assessed in two studies, with seven (78%) reporting favorable shifts toward stronger anti-vaping norms. These findings suggest differential responsiveness across cognitive-affective domains, with greater consistency in normative re-alignment than in perceived vulnerability measures.

### Acceptability and engagement

All twelve studies reported participant acceptability or engagement outcomes, assessed through satisfaction scales, usability scores, or qualitative feedback. Seven studies reported participant favourabble acceptability or engagement outcomes, assessed through satisfaction scales, usability scores, or qualitative feedback. These results confirm the feasibility and user receptiveness of digital and gamified interventions for youth audiences.

### Overall vote count synthesis

Across all constructs and studies, a total of 40 individual outcome votes were recorded. Of these, 75% (n=30) indicated favorable intervention effects, and 25% (n=10) showed no observable change. The overall synthesis indicates predominant positive psychosocial effects across knowledge, perceived risk, and social norms, with moderate gains in refusal self-efficacy and acceptability outcome.

### Summary of risk-of-bias assessment

Overall, global quality ratings ranged from weak to strong (Table 5). Six studies^26-29,32,33^ (50.0%) demonstrated strong methodological quality, two^30,35^ (16.7%) were moderate, and four^31,34,36,37^ (33.3%) were weak. Across individual domains, selection bias was low in eight studies (66.7%), while four (33.3%) showed higher risk due to unrepresentative samples or low participation rates. In terms of study design, seven studies (58.3%) were rated strong, four (33.3%) moderate, and one (8.3%) weak, reflecting variability in the use of experimental and quasi-experimental approaches. Control for confounding factors was strong in seven studies (58.3%), moderate in two (16.7%), and weak in three (25.0%), with weaker studies lacking sufficient adjustment for baseline characteristics. Blinding procedures were inconsistently applied, with four studies (33.3%) rated strong, four (33.3%) moderate, and four (33.3%) weak, primarily due to lack of concealment among participants or outcome assessors. Data collection methods were generally robust: eight studies (66.7%) used validated and reliable instruments, while three (25.0%) were moderate and one (8.3%) weak due to incomplete reporting of psychometric validation. Participant retention was satisfactory in most studies, with eight (66.7%) rated strong and four (33.3%) weak because of attrition exceeding 20% or incomplete reporting of follow-up.

**Table 5:** Summary of quality assessment using the Effective Public Health Practice Project tool

*Study*	*Selection* *bias*	*Study* *design*	*Confounders*	*Blinding*	*Data* *collection* *methods*	*Withdrawals and* *dropouts*	*Global rating*
Khalil et al.^26^ 2024	1	1	1	2	1	2	Strong
Nyman et al.^27^ 2024	2	1	1	1	1	2	Strong
Miller et al.^37^ 2022	3	2	1	3	2	1	Weak
Miley et al.^36^ 2022	3	2	3	3	3	2	Weak
Greene et al.^35^ 2021	2	2	2	3	1	2	Moderate
Guo et al.^31^ 2021	3	2	3	2	1	1	Weak
Weser et al.^30^ 2021	3	1	1	2	1	1	Moderate
Weser et al.^34^ 2021	3	2	3	3	1	2	Weak
Bteddini et al.^28^ 2023	1	1	1	2	1	1	Strong
Hieftje et al.^32^ 2019	1	2	1	2	1	2	Strong
Pentz et al.^33^ 2019	1	2	1	2	1	1	Strong
Parisod et al.^29^ 2018	1	1	1	1	1	1	Strong

Overall, half of the included studies demonstrated strong methodological quality, particularly in selection bias, study design, and data collection domains. The domains of blinding and confounder control exhibited the greatest variability, representing the primary sources of potential bias across the evidence base.

## DISCUSSION

This systematic review summarized the evidence on the effectiveness of vaping prevention interventions among adolescents delivered through digital gamification. These findings highlight the potential of digital interventions to enhance e-cigarette prevention intervention.

### Theory approach

The theoretical frameworks employed across studies in this systematic review highlight the diverse, yet complementary approaches utilized in gamified interventions for vape prevention and cessation. Social cognitive theory^39^ and the theory of planned behavior were the most applied frameworks^28,30,32-34^. These theories focus on cognitive processes, intention formation, behavioral control, and social influences, align closely with the interactive and socially driven nature of gamified interventions. Prior research supports the effectiveness of these theories in predicting health behaviors and guiding interventions in diverse health domains, including physical activity and dietary behaviour^40,41^. Studies^27,29^ employing Bandura’s Self-Efficacy Theory demonstrated how gamification elements effectively enhanced user self-efficacy. Previous research aligns, where enhancing self-efficacy consistently improves intervention outcomes across various behavioral health interventions such as smoking cessation and physical activity promotion^42,43^.

The ARCS model of motivation approached^31^ the issue through intrinsic motivational drivers (attention, relevance, confidence, and satisfaction) uniquely emphasized intrinsic motivation factors essential for sustaining long-term engagement, an aspect often less explicitly addressed by theories like the theory of planned behavior or self-efficacy theory. Previous studies utilizing the ARCS model in educational contexts also report improved motivation and sustained engagement, suggesting its potential for broader application in health interventions^44,45^.

Khalil et al.^26^ integrated multiple theoretical perspectives, including social learning theory, the health belief model, empowerment theory, and the transtheoretical model. Comparable multi-theoretical approaches have been effectively implemented in other behavioral health interventions, showing enhanced adaptability to diverse populations and complex behavior change contexts such as chronic disease self-management and obesity prevention^37,38^.

Despite these theoretical differences, convergences exist in terms of behavioral prediction and intervention strategies, specifically targeting individual cognitive appraisal, social influence, and perceived competence. However, critical differentiation lies in methodological implementation and targeted psychological components. Studies using the theory of planned behavior and social cognitive theory emphasized social interaction, observational learning, and normative feedback mechanisms^28,30,34^, effectively leveraging social dimensions inherent in gamification. Conversely, self-efficacy and ARCS model studies concentrated on individual motivation and competence through tailored feedback and personalized challenges.

An essential insight from this systematic review indicates that while multiple theoretical frameworks can effectively inform gamified interventions, integrating complementary theories may offer greater flexibility and comprehensive behavior change strategies.

### Intervention approach

The variability in intervention implementation highlights the complexity of vaping prevention research. Diverse settings introduce complexities due to differences in target populations, available resources, and contextual factors influencing adolescent behaviour^46^. School-based programs^47^ offer a broader reach, potentially capturing a larger proportion of at-risk youth, but less individualization compared to after-school or tailored interventions. These tailored approaches may be more effective for specific subgroups with unique needs or risk profiles, albeit with a narrower reach.

Physical versus online/remote delivery impacts accessibility, engagement, and the potential for personalized feedback. Online interventions improved accessibility and have wider dissemination, overcoming geographical barriers and resource limitations^48^. However, their effectiveness depended on digital literacy and consistent technology access, potentially exacerbating existing health disparities. Moreover, the lack of face-to-face interaction may diminish engagement and limit opportunities for personalized feedback and social support, crucial elements in behavior change interventions. Physical interventions were engaging but faced logistical constrained.

The range from 15-minute sessions to an hour, and from one-time to multiple sessions over weeks, introduces substantial variation in exposure and reinforcement. Brief interventions may raise awareness, but sustained change likely requires more intensive engagement^33,49^. Follow-up periods of up to nine months^35^ are helpful but may still be insufficient to capture the long-term impact on behaviour^47^. Given the dynamic nature of adolescent behavior and various factors influencing vaping habits, long-term follow-up is essential to ascertain the durability of intervention effects and identify potential relapse patterns^50^. Vaping habits can evolve significantly over time due to factors such as peer influence, exposure to marketing, and changes in regulatory policies^51^.

### Effectiveness

The evidence suggests that gamification and serious game can be a promising approach to e-cigarette prevention among youth. Digital gamified interventions can effectively increase knowledge, risk perception, and self-efficacy related to e-cigarette use, which is crucial for prevention. However, most studies measured short-term outcomes due to a short follow-up survey.

A consistent finding across multiple studies is that gamified interventions increased knowledge about tobacco and e-cigarettes. These findings suggest that gamification can be an engaging and effective way to educate young people about the risks and facts associated with vaping. However, it is crucial to acknowledge that knowledge alone may not be sufficient to deter vaping^52^. For instance, the Storm-Heroes intervention significantly increased tobacco knowledge at both post-intervention and follow-up assessments. Similarly, Catch Mybreath and Smokescreen demonstrated significant improvements in general knowledge and specific knowledge about e-cigarettes.

Beyond knowledge, gamification appears to influence risk perception and beliefs about e-cigarettes. One study found that a social game was more effective than a non-social game in increasing perceived risk of vaping, conventional knowledge, and tobacco knowledge. This finding is in agreement within tobacco context^53^. This demonstrates the potential of social gamification to leverage peer influence and social norms to shape attitudes towards vaping. The finding that beliefs mediated the relationship between knowledge and intentions to use e-cigarettes further demonstrates the importance of addressing beliefs in prevention efforts. The exact mechanisms by which gamified interventions alter beliefs and risk perceptions require further investigation.

Gamified approaches inherently leverage the motivational aspects of games to engage young people in learning about e-cigarettes. This is particularly important given the challenges of motivating adolescents to engage with traditional educational methods. The immediate effect of intention which was measured by Guo et al.^31^ showed relevance, satisfaction, and perceived persuasiveness significantly influenced the intention to abstain from smoking, suggesting learning motivation. ARCS motivation model has shown to be effective in improving knowledge, self-efficacy, and skills performance of nursing students delivered through 3D mobile application^54^. ARCS also has been used in a mobile augmented reality (MAR) application supporting teaching activities in interior design^55^. A meta-analysis revealed ARCS model of motivation has a large effect size on student learning achievement^49^. The Self-Determination Theory is an alternative theory that is often used as an intervention strategy for learning motivation promotion. The Self-Determination Theory assumes that individuals’ motivations are determined by their perceptions of autonomy, competence, and relatedness^56^.

Self-efficacy, the belief in one’s ability to succeed, is a critical factor in behavior change^39^. The ‘Fume’ health game improved smoking refusal self-efficacy, suggesting gamification can empower young people to resist the urge to vape. While the ‘Invite VR Only’ study did not show significant changes in self-efficacy, participants maintained a consistently high level of self-efficacy throughout the study, indicating VR games may be useful in reinforcing existing beliefs, hence important to incorporate how to maintain it over time.

Social games are more effective in increasing perceived risk, highlighting the harness of peer influence and social norms. Social elements can create opportunities for young people to learn from and support one another in resisting e-cigarette use^26^. Interventions that incorporate peer interaction and social support may be more effective in promoting long-term behavior change.

Long-term follow-up reveals that while the overall perceived likelihood of e-cigarette use remained low, there were no significant changes between groups. While the game might initially reduce intentions, its effect may fade over time, potentially due to other factors influencing the participants. External influences (e.g. peer pressure marketing, accessibility) likely play a more dominant role in shaping long-term intentions and behaviour^51^. Providing booster sessions or ongoing engagement to sustain the effects of gamified interventions is needed.

### Strengths and limitations

This review has several strengths. It is the first to synthesize the evidence on digital gamification interventions specifically targeting e-cigarette use prevention among adolescents, with a strong emphasis on theoretical underpinnings. The methodology adhered to PRISMA 2020 guidelines and SWiM, was registered in PROSPERO, and included a comprehensive search across nine databases. Dual independent screening, data extraction, and quality assessment further enhanced methodological rigor. Despite promising findings, it is important to acknowledge the limitations related to both the body of evidence and the review process itself. First, the majority of included studies assessed only short-term outcomes, typically within weeks to a few months after the intervention. This restricts understanding of the long-term effectiveness of gamified interventions on sustained behavior change. This suggests that gamification alone may not be sufficient to change behavior and may need to be combined with other prevention strategies. The outcome relies on self-reporting, which is subject to biases such as social desirability bias (participants may underreport their likelihood of experimenting to align with perceived expectations). The heterogeneity of intervention designs including differences in platform (e.g. VR, web-based, hybrid), intensity, and theoretical underpinnings, makes cross-study comparisons difficult. Finally, most studies were conducted in high-income countries, potentially limiting generalizability to low- and middle-income contexts where adolescent vaping dynamics and digital access may differ. The review included only English-language articles, potentially introducing language bias by excluding relevant studies published in other languages. Due to the heterogeneity of study designs and outcome measures, a meta-analysis was not feasible, preventing pooled effect estimation.

### Implications for practice and policy

The findings from this review offer actionable insights for public health practitioners, educators, and policymakers. First, incorporating gamification into school-based and digital health programs may enhance the reach and appeal of e-cigarette prevention efforts among adolescents. Interventions grounded in established behavioral theories and designed with user engagement in mind such as through feedback mechanisms, peer interactions, and narrative elements are likely to have stronger impact. Second, to ensure effectiveness and equity, gamified programs must be accessible across diverse socioeconomic and cultural contexts. This calls for collaboration between health agencies, educators, developers, and youth themselves to ensure cultural sensitivity, affordability, and relevance. Third, policymakers should consider supporting digital health innovations, including gamified prevention tools, through integration with national adolescent health strategies and funding mechanisms. Emphasis should also be placed on rigorous program evaluation frameworks that measure both short- and long-term outcomes. Finally, regulatory measures, such as restrictions on marketing flavored vape products to youth should complement educational interventions. A multisectoral approach that combines gamified digital tools with supportive policies can offer a more comprehensive response to the vaping epidemic among adolescents.

### Future research

Future research should explore the optimal design features of gamified interventions, such as the inclusion of social elements, personalized content, and adaptive challenges. It would also be valuable to investigate the long-term effects of gamification on e-cigarette use and to identify the specific mechanisms through which gamification influences behavior change. Integrating learners’ experiences into the development of learning material can improve learning effectiveness. Future research should focus on identifying the mechanisms through which interventions produce behavior change. How interventions can be designed to foster autonomy, competence, and relatedness, and how these factors influence motivation and behavior change should also be explored.

## CONCLUSIONS

The review identified a limited number of studies that met the inclusion criteria, reflecting the emerging nature of this field. The included studies varied in terms of study design, intervention characteristics, and outcome measures, but collectively provided evidence to support the potential of digital gamification as a promising approach to prevent vaping among adolescents. To maximize the effectiveness, it is essential to carefully design interventions based on established behavior change theories. Incorporating social elements and personalized content can further enhance engagement and effectiveness. Addressing core beliefs and perceptions surrounding e-cigarettes is equally vital, and these interventions should ideally be integrated with broader prevention strategies, such as educational initiatives, policy changes, and community-based programs. Continuous evaluation and adaptation are essential to ensure relevance and efficacy in the face of an evolving e-cigarette landscape, and to maximize the potential of gamification in shielding young individuals from the detrimental effects of e-cigarettes.

## Supplementary Material



## Data Availability

The data supporting this research are available from the authors on reasonable request.
