# Supplementary material for: Digital gamification and serious games for preventing e-cigarette use in adolescents: A systematic review without meta-analysis (SWiM)
Source: Tob Induc Dis. 2026 Jul 16;24:10.18332/tid/214070. doi: 10.18332/tid/214070 (PMC13377530; doi:10.18332/tid/214070)
Supplement: Supplementary file 1 [file TID-24-110-s1.pdf]

Tobacco Induced Diseases

Supplementary file

© 2026 Hasan S.I. et al.

DOI:
10.18332/tid/214070

The content has been provided by the author(s) and has not been reviewed, verified, or endorsed by European Publishing. It may not have undergone peer review. The views, opinions, and recommendations expressed are solely those of the author(s) and do not necessarily reflect the position of European Publishing. European Publishing accepts no responsibility or liability for any consequences arising from the use of, or reliance on, this content.

Section and Topic	Item #	Checklist item	Reported (Yes/No)
**TITLE**			
**BACKGROUND**			
**METHODS**			
**RESULTS**			
**DISCUSSION**			
**OTHER**			

From:

Section and Topic	Item #	Checklist item	Location where item is reported
**TITLE**			
**ABSTRACT**			
**INTRODUCTION**			
			–
**METHODS**			
			→ Study Screening
			–

Section and Topic	Item #	Checklist item	Location where item is reported
**RESULTS**			
			–
			–
**DISCUSSION**			
**OTHER INFORMATION**			

Section and Topic	Item #	Checklist item	Location where item is reported

From:

#1
ALL=(Electronic Nicotine Delivery Systems OR Vaping) Vaping – Electronic Nicotine Delivery Systems – E-cigarettes – E-cigarette – Electronic Cigarettes – Electronic Cigarette – Ends – Electronic Nicotine Delivery System – Electronic Nicotine Delivery Devices –

ALL=((adolescent OR child OR teen OR youth)) Children – Adolescents – Child – Adolescent – Adolescence – Youth –

ALL=(Gamification) Gamification – Game Elements – Serious Games – Game-based Learning – Gamification In Education – Gamified Learning –

#4
ALL=(prevention OR cessation) Prevention – Smoking Cessation – Primary Prevention – Secondary Prevention –
